# Self-assembled lipid-based nanoparticles for chemotherapy against breast cancer

**DOI:** 10.3389/fbioe.2024.1482637

**Published:** 2024-10-29

**Authors:** Shan Liu

**Affiliations:** ^1^ Department of Oncology, Songjiang Hospital Affiliated to Shanghai Jiao Tong University School of Medicine, Shanghai, China

**Keywords:** self-assembly, lipids, nanoparticles, breast cancer, chemotherapy

## Abstract

Self-assembled lipid-based nanoparticles have been shown to have improved therapeutic efficacy and lower toxic side effects. Breast cancer is a common type of malignant tumor in women. Conventional drugs such as doxorubicin (DOX) have shown low therapeutic efficacy and high drug toxicity in antitumor therapy. This paper surveys research on self-assembled lipid-based nanoparticles by categorizing them under three groups: self-assembled liposomal nanostructures, self-assembled niosomes, and self-assembled lipid–polymer hybrid nanoparticles. Subsequently, the structural features and operating mechanisms of each group are summarized individually along with examples of representative drugs from each group.

## 1 Introduction

Breast cancer is a common type of malignant tumor among women (Ashkbar et al., 2020). Between 2008 and 2020, the number of female breast cancer cases increased from 1.38 million to 2.25 million (Tseu and Kamaruzaman, 2023; Riis, 2020). The main factors that induce breast cancer include heredity, menstruation, fertility, and living habits (Kshivets, 2023; Jemal et al., 2011; Britt et al., 2020)}. The 5-year survival rate for breast cancer is over 90% (Riis, 2020); however, breast cancer remains the second leading cause of tumor-related deaths among women worldwide (Yang et al., 2021). Breast cancer treatments have now entered the era of precision medicine (Sarhangi et al., 2022). Traditional chemotherapeutic drugs have low bioavailability and poor efficacy owing to their poor selectivity toward cancer cells, poor solubility, low stability, and high side effects (Yang et al., 2021; Qiao et al., 2020). In addition, the ability of chemotherapeutic drugs to penetrate cancer cells is limited, often resulting in drug resistance (Tredan et al., 2007; Grantab et al., 2006; Ning, 2022). Thus, improving the drug concentration at the cancer site and reducing the side effects are important directions of current research (Lim and Ma, 2019).

Self-assembled nanoparticles loaded with drugs constitute a class of medications characterized by their nanoscale structures. These nanoparticles are formed by self-assembly and incorporate small molecules of chemotherapeutic or macromolecular drugs, such as proteins and nucleic acids. During this process, they may experience interactions involving hydrogen bonds, electrostatic forces, van der Waals forces, and other related forces (Song et al., 2012). Currently, most nanoparticles used in clinical practice have diameters in the range of 1–200 nm. Upon entering the body, the nanoparticles maintain balanced surface charges to prevent infiltration of the encapsulated drugs into the surrounding tissues (Song et al., 2012; Hoshyar et al., 2016; Cheng et al., 2020; von Roemeling et al., 2017; Schwartz, 2017; Xie et al., 2017). Various self-assembled nanoparticles have been synthesized from carbohydrates, nucleic acids, peptides, and other biomaterials for biomedical and pharmaceutical applications (Dahiya and Dahiya, 2021; Fan et al., 2016; Kaur et al., 2023; Jain et al., 2015). The comprehensive treatment of cancer using self-assembled nanoparticles not only enhances the survival rates but also mitigates the risks associated with low local drug utilization and excessive systemic adverse drug reactions (Pérez-Herrero and Fernández-Medarde, 2015; Sabit et al., 2022; Kitsios et al., 2023).

Existing research classifies self-assembled nanocarriers into six categories for various medical applications as lipid-based nanoparticles (Lasic, 2019; Puri et al., 2009; Sabahi, 2023), polymeric nanostructures, carbon-based nanoparticles, ceramic nanostructures, biological nanoparticles, and micelles (Lasic, 1996; Torchilin, 2006; McClements, 2012; Allen and Cullis, 2013; Cabral and Kataoka, 2014; Menjoge, 2010; Varma et al., 2020; Da, 2021; Xie and Pedrielli, 2023). Self-assembled lipid-based nanoparticles offer numerous advantages, including versatility, biocompatibility, controlled drug-release capabilities, enhanced stability, and targeted delivery potential. Some representative drugs in this category include DOX-Lip, pegylated liposomal doxorubicin (PLD), nitric oxide (NO)-donor-loaded bioinspired lipoprotein system (NO-BLP), albumin-bound paclitaxel lipid nanoparticles (ABPLN), and liposomal daunorubicin.

This review comprehensively introduces three distinct categories of self-assembled lipid-based nanoparticles, namely self-assembled liposomal nanostructures, self-assembled niosomes, and self-assembled lipid–polymer hybrid nanoparticles (LPHNPs). We describe the structures and compositions of these nanoparticles; furthermore, we provide a summary of the related drugs and current research status by analyzing their advantages and disadvantages, thereby enabling discussion of the extent to which self-assembled lipid-based nanoparticles can optimize chemotherapy in breast cancer (as illustrated in Table 1).

**TABLE 1:** Advantages and disadvantages of typical self-assembled lipid-based nanoparticles.

Taxonomic category	Advantages	Disadvantages	References	Research objects	Representative drugs
Self-assembled liposomal nanostructures	Enhance drug biocompatibility; targeted drug delivery; deep tumor penetration and release	Liposomes are thin and fragile with poor storage stability	Pestalozzi et al. (1992) and Pestalozzi et al. (1995); Poujol et al. (1999); Makowski et al. (2019); Chowdhury et al. (2020); Wang et al. (2020); Yang et al. (2022)	HER-2+ breast cancer cells; 4T1 cells	Pegylated liposomal DOX; liposome-complexed mitoxantrone
Self-assembled niosomes	Better economy; more stable; higher encapsulation rate	Combination toxicity unaltered	Liu et al. (2017); Assali et al. (2022)	MCF-7 breast cancer cells; Peppas–Sahlin model	Liposomal daunorubicin
Self-assembled lipid–polymer hybrid nanoparticles	Repeatability; stability; controllable; tumor targeting; high drug efficacy	Solvent toxicity; toxic products; limited drug-capture capacity	Ruttala and Ko (2015b, 2015a); Stylianopoulos et al. (2018); Chowdhury et al. (2020); Martin et al. (2019); Wu et al. (2023)	MCF-7 breast cancer cells; 4T1 cells	Albumin-bound paclitaxel lipid nanoparticles

## 2 Research advancements on using diverse self-assembled lipid-based nanoparticles in chemotherapy for breast cancer

The Guidelines for Clinical Diagnosis and Treatment of Advanced Breast Cancer in China (2023 Edition) clearly state that anthracyclines, including epirubicin and doxorubicin (DOX) (Devi et al., 2023; Hossain et al., 2023), are the preferred first-line treatment drugs for breast cancer. Nevertheless, the main challenge is reducing the toxicity of anthracyclines, which significantly impacts the clinical management of breast cancer. As a representative drug delivered by self-assembled lipid-based nanoparticles, such as PLD/ABPLN, it enhances drug utilization while mitigating the toxic side effects.

### 2.1 Compositions of three kinds of self-assembled lipid-based nanoparticles

Through *in vitro* experiments, animal studies, and clinical trials, it has been found that self-assembled lipid-based nanoparticles can provide increased drug concentration while minimizing the toxicity and side effects. In this context, the three self-assembled lipid-based nanoparticles used in chemotherapy against breast cancer are self-assembled liposomal nanostructures, self-assembled niosomes, and self-assembled LPHNPs.

#### 2.1.1 Self-assembled liposomal nanostructures

Liposomes are nanomaterials used for drug delivery (Bhattacharya et al., 2022; Zylberberg et al., 2017). Nanoparticles prepared as liposomes were among the first to be applied in clinical drug delivery systems, offering advantages such as biocompatibility and degradability (Torchilin, 2005; Allen and Cullis, 2013; Al-Jamal and Kostarelos, 2011). Liposomes also have disadvantages, such as being thin, being fragile, and having poor storage stability. Bangham’s phospholipid experiment in the 1960s revealed that hydrophilic groups of self-assembled liposomes exposed to water with hydrophobic groups hidden inside facilitate liposomal delivery systems through molecular interactions. The amphiphilic components of the liposomes self-assemble in aqueous media spontaneously to form stable structures. Self-assembled liposomal nanostructures can adjust their performances through alterations to the composition and surface (Das et al., 2013; Logigan et al., 2024; Ewert et al., 2023; Stephanopoulos et al., 2013; Genio et al., 2022).

The cytotoxic drug DOX can stop the proliferation of cancer cells by inhibiting the syntheses of topoisomerase II and nucleic acids. DOX combined with liposomes can reduce the side effects of DOX itself and overcome the limitations of clinical medications (Makowski et al., 2019). Rahman et al. (1989) at Georgetown University found that liposomes with encapsulated DOX could inhibit DNA synthesis in human breast cancer (MDA-435) cells. Upon addition of a monoclonal antibody against human laminin receptor to the surface of the liposomal DOX, the binding to the target cells was found to have increased tenfold. Since the mid-1990s, long-acting DOX (also known as Doxil in the United States and Caelyx in Europe) encapsulated in polyethylene glycol (PEG) liposomes has been marketed and applied to metastatic breast cancer (Waterhouse et al., 2001; Duggan and Keating, 2011).

**FIGURE 1:**
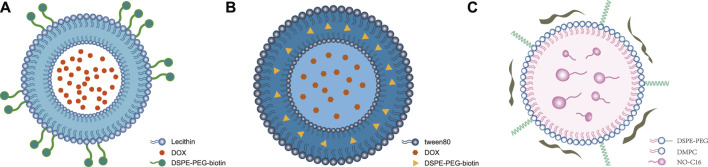
Structures of self-assembled lipid-based nanoparticles. **(A)** Self-assembled liposomal nanostructures: modified DSPE-PEG-biotin (streptavidin, biotin, PEG, and macrophage synthesis) on a double-layer structure to encapsulate DOX to form DOX-Lip (Yang et al., 2022) ⓒ 2022, American Chemical Society. **(B)** Self-assembled niosomes: Tween-80-conjugated niosomes with cholesterol encapsulate DOX and D-limonene in lipid-based nanoparticles. **(C)** Self-assembled lipid–polymer hybrid nanoparticles: NO-donor-loaded bioinspired lipoprotein system (NO-BLP) was constructed using 1,2-dimyristoyl-sn-glycero-3-phosphatidylcholine (DMPC), DSPE-PEG 2000, NO-C16, and ApoA1 mimetic peptide (Wu et al., 2023) ⓒ 2023, American Chemical Society.

Unlike DOX, which inhibits lipid peroxidation and induces the formation of free radicals, mitoxantrone is superior in terms of its acute toxicity and cross-resistance, especially in the treatment of metastatic breast cancer (Durr et al., 1983; Pestalozzi et al., 1992). Poujol et al. (1999) found that in patients with multidrug-resistant breast cancer, certain compounds could alter the P-glycoprotein function through plasma membrane stabilization and modulate multidrug resistance in human cancers based on their lipid compositions. Mitoxantrone-loaded liposomes have improved drug safety but have not significantly improved the antitumor abilities of the drug. Moreover, they do not show evident advantages over self-assembled liposomal nanostructures loaded with DOX. Therefore, Chowdhury et al. (2020) continued their research on DOX in self-assembled liposomal nanostructures; they used phosphatidylcholines, cholesterol, and 1,2-distearoyl-sn-glycero-3-phosphoethanolamine with conjugated methoxy PEG (DSPE-mPEG) to develop a lipid–polymer coating for DOX. Their experiments on HER-2-positive breast cancer cells showed increased uptake of DOX with a lower IC_50_ value. It was observed that the presence of liposomes in the HER-2-positive MCF-7 and SKBR-3 breast cancer cells increased the uptake and targeted delivery of DOX. Yang et al. (2022) modified DSPE-PEG-biotin, conjugated streptavidin (STA), biotin, and PEG on double-layer phospholipids to encapsulate DOX and form DOX-Lip (Figure 1A). Furthermore, DOX-Lip was connected to macrophages to form the macrophage liposome (MA lip). A study on a triple-negative breast cancer cell line (4T1 cells) showed that MA lip was conducive to the migration of DOX into the deep cells of the tumor and its release into the deep regions of the tumor.

It was shown that DOX induces the immune cells to activate antitumor immunity (Galluzzi et al., 2020), and it was also confirmed that macrophages actively migrate into hypoxic areas within tumors (Chaturvedi et al., 2014; Germain and Kastenmüller, 2010). Existing research shows that self-assembled liposomal nanostructures are formed using single-layer or double-layer lipid outer membranes composed of substances such as phospholipids, cholesterols, DSPE-mPEG, and lipids encapsulating chemotherapeutic drugs like mitoxantrone or DOX with or without surface modification by DSPE-PEG-biotin (Figure 1A) (Chowdhury et al., 2020; Yang et al., 2022; Pestalozzi et al. (1992) and Pestalozzi et al. (1995); Wang et al., 2020). The representative drugs PLD and liposome-complexed mitoxantrone were also shown to have improved safety.

#### 2.1.2 Self-assembled niosomes

Niosomes are nanovesicles of non-ionic surfactants that promise efficient drug delivery by encapsulating hydrophilic and hydrophobic drugs. Riccardi et al. (2018) used the AS1411 aptamer to target cancer cell nucleolin, whereby the drugs were delivered directly to the breast tumors with reduced toxicity and enhanced treatment efficacy. Liu et al. (2017) studied liposomal daunorubicin as a representative drug of self-assembled niosomes to enhance its retention time in tumor tissues. Wheat germ agglutinin (WGA)-modified daunorubicin antiresistant liposomes were produced by the thin-film hydration method using coated WGA (Li et al., 2014, Li et al., 20142015) with a lipid composition of error-producing condition (EPC)/cholesterol/DSPE-PEG 2000. In tumor-bearing mice with MCF-7/ADR cells, the WGA-modified daunorubicin antiresistant liposomes were found to retain daunorubicin in the tumor tissues for 24 h and significantly reduce the size of the tumor cells; free daunorubicin stayed in the tumor tissues for only a short time. However, liposomal daunorubicin also has some limitations, such as its high molecular weight. Although liposomal daunorubicin has low toxicity even with its lengthy retention time in living tissues, further studies are needed to assess whether there will be toxicity accumulation. Kulkarni and Rawtani (2019) from the American Pharmaceutical Association used the Box Behnken design to study MCF-7 breast cancer cells *in vitro*. They found that niosomes containing the lipophilic drug tamoxifen and hydrophilic drug DOX had 70% encapsulation efficiency and could be stable for up to 6 months under refrigeration at 4°C; this composition shows increased encapsulation efficiency and stability simultaneously. Although blank niosomes are non-toxic to normal cells, the niosomes used in this study could not reduce the cumulative toxicity of the drugs in combination therapy for the time being. Assali et al. (2022) used the Peppas–Sahlin model and found that niosomes containing Tween 80 with cholesterol and encapsulating DOX as well as D-limonene exhibited higher stability; *in vitro* experiments also showed that the release of DOX was more stable (Figure 1B). However, the study did not identify the protective effects of D-limonene combined with DOX on the heart, indicating that further research is needed. 

Unlike self-assembled liposomal nanostructures using DSPE-PEG 2000 as the medium, self-assembled niosomes contain Tween 80 as the medium. As shown in Figure 1B (Assali et al., 2022; Liu et al., 2017), liposomal daunorubicin as a representative drug controls the progress of breast cancer by increasing the retention time of daunorubicin in the tumor tissues.

#### 2.1.3 Self-assembled LPHNPs

Self-assembled LPHNPs have advantages as nanocarriers for the delivery of anticancer agents (Pang et al., 2020; Martinelli et al., 2019; Govindan et al., 2023); these benefits include compositions of diverse synthetic drugs, simple and repeatable synthesis process, and good stability (Du et al., 2019). These nanoparticles also enable individualized treatments for tumor patients through different chemical modifications (Kumar et al., 2020). However, owing to the use of organic solvents during synthesis, the LPHNPs exhibit shortcomings, such as generating toxic byproducts after *in vivo* degradation and limited capacity to capture drugs (Ulbrich et al., 2016). Rao and Prestidge (2016) encapsulated polymer particles in a lipid shell to synthesize LPHNPs and explored their feasibility for oral administration. LPHNPs mainly comprise an external lipid shell encapsulating biodegradable hydrophobic polymers, with the inner core serving as the primary carrier of lipid-soluble drugs (Shi et al., 2015; Li, 2024; Dehaini et al., 2016; Wu et al., 2020; Sengel-Turk et al., 2021). LPHNPs have strong lipid-based drug-loading capacities and can affect the drug release rates (Bakar-Ates et al., 2020; Klibanov et al., 1990; Hamdi et al., 2020). Moreover, LPHNPs possess biomimetic properties, biocompatibility, ideal drug-release characteristics for polymer nanocarriers, and the ability for various surface chemical modifications (Al-Jipouri et al., 2023; Shi et al., 2014; Tahir et al., 2020). Zhang et al. (2021) prepared nanoparticles using the Michael-type step polymerization (Lynn and Langer, 2000), where a mixed lipid shell comprising DSPE-PEG 2000, FA-DSPE-PEG 2000, and lecithin encapsulates a degradable poly β-amino ester (PBAE) carrying docetaxel (DTX) to form PBAE/DTX nanoparticles. FA-targeted phospholipid monolayers form the shell of the FA/PBAE/DTX nanoparticles. The study of 4T1 breast cancer cells showed that compared to free DTX or PBAE/DTX nanoparticles, FA/PBAE/DTX nanoparticles had significantly enhanced intracellular uptake efficiency and cytotoxicity. The modification with FA enhances the tumor-targeting ability of the polymeric lipid nanoparticles. Finally, FA/PBAE/DTX nanoparticles for drug delivery enhanced the antitumor effects and had less systemic toxicity in 4T1 breast cancer cells. A sunitinib-loaded self-nanoemulsifying formulation has been reported to have better antitumor activity against MCF-7 breast cancer cells (Nazari-Vanani et al., 2017; Adams and Leggas, 2007; Alshahrani et al., 2018). Further studies are being conducted on sunitinib malate (SM) in 28 ongoing clinical trials (Chen et al., 2017). Another study on MCF-7 cells involved the use of lecithin as a stabilizer along with emulsification-solvent evaporation technology (Ahmed et al., 2022; Anwer et al., 2021). SM, Lipoid 90H, and chitosan have been combined to form LPHNPs; by adjusting the concentration of chitosan, the composition of the nanoparticles can be altered. Accordingly, four formulations (SLPN1–SLPN4) were developed and tested separately on MCF-7 cells. The results showed that SLPN4 significantly enhances the release and accessibility of SM in MCF-7 cells and that SM-loaded LPHNPs may be a promising option for cancer treatment.

Existing clinical studies have shown that albumin-bound paclitaxel increases the safety of paclitaxel over traditional paclitaxel for HER-2-positive, weak-positive, or HER-2-negative patients (Shi et al., 2023; Futamura et al., 2023). However, no significant changes have been found in the survival rates of breast cancer patients. Ruttala and Ko (2015b) improved the antitumor effects of albumin-bound paclitaxel nanoparticles (APN); the lipid–liposome bilayer was coated with albumin paclitaxel to prepare liposome-encapsulated APN (i.e., L-APN or ABPLN). *In vitro* tests then showed that L-APN could significantly improve the stability of paclitaxel and enhance the cytotoxic activity of APN in MCF-7 cells; it was also found via *in vitro* studies that when curcumin and APN are codelivered to MCF-7 cells, the presence of the liposomes enhances the synergistic antitumor ability of curcumin and albumin-bound paclitaxel (Ruttala and Ko, 2015a).

Compared to self-assembled LPHNPs coated with APN, liposomal nanoparticles coated with curcumin and albumin-bound paclitaxel exhibit greater cytotoxicity and superior anticancer effects on breast cancer cells. Based on these findings, scholars from Fudan University in Shanghai constructed a NO-BLP (Wu et al., 2023) composed of 1,2-dimyristoyl-sn-glycero-3-phosphatidylcholine (DMPC), DSPE-PEG 2000, glutathione (GSH)-sensitive NO donor agent (NO-C16), and an apolipoprotein A1 (ApoA1) mimetic peptide (Figure 1C) (Stylianopoulos et al., 2018; Martin et al., 2019). In mice carrying 4T1 tumors of breast cancer, NO-BLP can effectively accumulate at the tumor site and release active NO molecules to normalize the disordered tumor vessels, promoting intratumoral administration and chemotherapy using APN. Self-assembled lipid–polymer hybrid nanoparticles can modify the tumor microenvironment and enhance the efficacy of chemotherapeutic drugs by releasing NO into the tumor cells. Therefore, NO-BLP improves the efficacy of APNs. However, as this study was conducted in animals, only the antitumor effects were evaluated while the drug safety was not assessed; thus, drug safety evaluations are necessary for further clinical trials.

### 2.2 Action mechanisms of self-assembled lipid-based nanoparticles

#### 2.2.1 Prolonging the retention times of chemotherapeutic drugs in tumor tissues and activating strong immune responses

**FIGURE 2:**
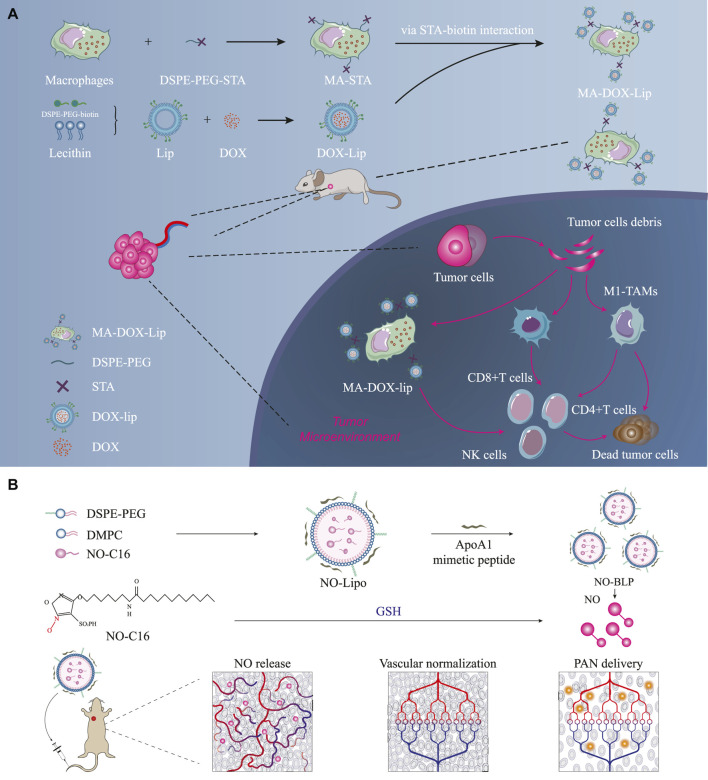
Prolonging the retention times of chemotherapeutic drugs in tumor tissues and activating strong immune responses. **(A)** DOX-Lip links with macrophages through DSPE-PEG-STA interactions; mechanism of *in vivo* action of self-assembled lipid-based nanoparticles (Yang et al., 2022) ⓒ 2022, American Chemical Society. **(B)** Schematic illustration of self-assembled lipid-based nanoparticles acting on breast cancer tissues to restore normal blood supply (Wu et al., 2023) ⓒ 2023, American Chemical Society. Here, DSPE-PEG denotes 1,2-distearoyl-sn-glycero-3-phosphoethanolamine-poly (ethylene glycol); DMPC denotes 1,2-dimyristoyl-sn-glycero-3-phosphatidylcholine; PAN denotes paclitaxel nanoparticles; NO-BLP denotes NO-donor-loaded bioinspired lipoprotein system.

Most recent studies show that self-assembled lipid-based nanoparticles can prolong the residence times of chemotherapeutic drugs in tumor tissues (Makowski et al., 2019; Chowdhury et al., 2020; Yang et al., 2022). Yang et al. (2022) showed that DOX-Lip links with macrophages through DSPE-PEG-STA interactions (Figure 2A) and deeply penetrates the tumor cells to release DOX, thereby activating robust immune responses through the CD4^+^/CD8^+^/NK cells. MA-DOX-Lip effectively inhibited the tumor growth model of 4T1 triple-negative breast cancer in mice and improved the killing rate of the tumor cells (Figure 2A). Recent studies have also proven that the presence of liposomes improves the utilization rates of chemotherapeutic drugs and their safety. However, some studies have shown that increasing the utilization of chemotherapeutic drugs has no apparent advantages for prolonging the overall survival (OS) of cancer patients.

#### 2.2.2 Self-assembled lipid-based nanoparticles restore regular blood supply to tumor tissues

Although traditional research efforts have prolonged the retention times of chemotherapeutic drugs in tumor tissues, they have not significantly prolonged the OS of breast cancer patients because of the influence of the tumor microenvironment on antitumor efficacy (Tredan et al., 2007). Wu et al. (2023) prepared NO-BLP loaded with GSH-activated NO donors to normalize tumors, improve the delivery efficiency of APN in the tumors, change the tumor microenvironment by restoring regular blood supply to the tumor tissues, and sequentially treat tumors (Figure 2B). The tumor inhibition rate for the NO-BLP + PAN treatment group was 81.03%, and the tumor sizes were only 29.89% for the NO-BLP group and 39.85% for the PAN group (Wu et al., 2023).

Changes in the tumor microenvironment are likely to promote the effects of chemotherapeutic drugs and reduce drug resistance (Tredan et al., 2007). Unlike traditional research on activating the immune responses, research on self-assembled lipid-based nanoparticles offers an alternative approach by which the nanoparticles change the tumor microenvironment to improve the drug utilization rate. Research on restoring regular blood supply to the tumor tissue with nanomaterials can improve the killing rates of tumor cells using chemotherapeutic drugs through the changes to the tumor microenvironment. However, such research efforts are still within the scope of animal research. Although animal research proves the safety and reliability of a drug—supported by the weights of the tumor-bearing mice and histological examination results of their main organs—there is a lack of relevant clinical trials, resulting in inadequate evaluations of the safety and antitumor effects. The extent to which NO-BLP improves the effectiveness of other liposomal chemotherapeutic drugs remains an open research question.

### 2.3 Current clinical research progress on self-assembled lipid-based nanoparticles

Existing clinical antitumor treatments widely use liposomal adriamycin, and clinical trials have improved its safety, reduced the toxic and side effects, and ensured a smoother process for chemotherapy, thereby prolonging the progression-free survival (PFS) (China, 2023). Unlike the results of *in vitro* studies and animal experiments, the aforementioned drugs have not improved the OS times of breast cancer patients over 5 and 10 years, which may be related to insufficient sample sizes and short research times (Gucalp et al., 2018; Fujiwara et al., 2019; Pestalozzi et al., 1992, 1995; Fabi et al., 2020). Pestalozzi et al. (1992); Pestalozzi et al. (1995) found that in the phase II study of liposome-complexed mitoxantrone, although there was no significant improvement in the PFS over treatment with free mitoxantrone, there were reductions in the cardiac and hematological toxicities. To verify whether breast cancer patients could benefit from self-assembled liposomal nanostructures, O’brien et al. conducted a phase III study comparing the use of DOX and PLD as first-line treatments in 509 patients with metastatic breast cancer (Wang et al., 2020). PLD was formulated as photolipid bilayers coated with methoxy-PEG-encapsulated DOX. The OS rates of patients showed no differences for the two treatments (PLD over 21 months vs. DOX over 22 months); however, the pathological complete response (PCR) of the PLD group was significantly prolonged and cardiac toxicity was significantly reduced (hazard ratio [Hr] 1/4 = 3.16; 95% confidence interval [cI] 1/4 = 1.58–6.31; p<0.001 ). Research results from patients with clinical stage IIA–IIIc and lymph node position indicate that PLD is safer and more reliable than DOX in the combined treatment of metastatic breast cancer, and prolonging the PCR also reduces the cardiotoxicity of DOX. The main drawback of this study is that its sample size was too small and HER-2-positive cases were not grouped, so studies on a larger population would be needed in the future. Fabi et al. (2020) conducted a phase I study on HER-2-negative metastatic breast cancer patients and found that liposoluble DOX had improved drug safety and reduced chemotherapeutic drug toxicity.

Clinical experiments on PLD and liposomal mitoxantrone, which are some representative drugs used in self-assembled liposomal nanostructures, show that liposomal nanostructures can indeed increase drug safety and relatively improve drug utilization compared to free DOX/mitoxantrone. However, there are no apparent benefits in terms of drug resistance, especially in prolonging the survival of breast cancer patients.

Although self-assembled niosomes have produced good results *in vitro* and in animal experiments and liposomal daunorubicin used as their representative drug significantly reduced the growth rates of breast cancer cells in MCF-7/ADR-tumor-bearing mice, there are no clinical trials that clearly show that self-assembled niosomes can simultaneously improve the efficacy and safety of antitumor drugs. Liposomal daunorubicin as a representative drug used in self-assembled niosomes has improved the antitumor efficacy in chemotherapy because it prolongs the residence time of daunorubicin in the tumor tissues. However, the safety of relevant drugs and nanomaterials are still subject to verification. Compared with self-assembled liposomal nanostructures that improve the safety of antitumor treatments, self-assembled niosomes have more advantages in reducing the tumor growth rates; however, unlike the phase I/II study of self-assembled liposomal nanostructures, there are no available clinical studies of self-assembled niosomes, suggesting that breast cancer patients can significantly benefit from more studies on self-assembled niosomes. Furthermore, clinical experiments are needed to clarify whether self-assembled niosomes can achieve better antitumor effects in humans.

As a representative drug used in self-assembled LPHNPs, APN (or L-APN), ABPLN can improve the safety of chemotherapy and is more convenient for clinical application (Ruttala and Ko, 2015b; Meng et al., 2015). However, there is limited research on these drugs against breast cancer. *In vitro* research shows that NO-BLP or self-assembled LPHNPs have better anticancer effects and can further improve the antitumor effects in chemotherapy by changing the tumor microenvironment. A phase I study of APN also showed that self-assembled lipid-based nanoparticles could reduce the toxicity of chemotherapeutic drugs (Fabi et al., 2020). Compared with traditional free paclitaxel/DOX, liposome-encapsulated DOX combined with APN has significantly reduced toxicity. Nevertheless, as a phase I study involving only 12 patients, this approach needs improvement given the inadequate number of participants, samples, and data.

## 3 Conclusion

Nanomedicine as a field is still limited for the comprehensive treatment of breast cancer (Kim et al., 2007; Fabian et al., 2020; Gucalp et al., 2018; Malloizel-Delaunay et al., 2024; Wu et al., 2017), and clinical trials related to nanomedicine have also proved the importance of nanobioengineering for disease control in clinical tumor patients (Fujiwara et al., 2019; Mohammadpour and Majidzadeh, 2020; Hekmat et al., 2022). The presently available self-assembled lipid-based nanoparticles increase the drug utilization concentrations in tumor tissues as well as reduce the toxic and side effects of drugs, thereby improving the safety of tumor treatment.

Nowadays, chemotherapy as a treatment for breast cancer is limited by the toxic and side effects of the drugs used, which affects the treatment efficacy. Although the phase I studies conducted by Pestalozzi et al. (1992) and Pestalozzi et al. (1995), O’brien (Wang et al., 2020), and Fabi et al. (2020) lack adequate sample sizes, there is evidence that self-assembled liposomal nanostructures encapsulated with DOX or mitoxantrone can improve the safety of chemotherapy drugs as well as significantly reduced their toxic and side effects, especially in terms of cardiotoxicity. However, it is debatable whether improving the chemotherapy cycle of DOX can further prolong the OS times of breast cancer patients and must be verified with additional clinical studies. Moreover, *in vivo* and *in vitro* experiments have shown that the representative drugs used in self-assembled lipid-based nanoparticles, such as DOX-Lip, liposome-complexed mitoxantrone, ABPLN, and liposomal daunorubicin, significantly improve the safety of chemotherapy in the free state and reduce cardiotoxicity. At the same time, extant studies show that DOX-Lip, liposome-complexed mitoxantrone, and ABPLN mainly enhance safety by reducing the toxic and side effects of chemotherapeutic drugs, with no significant changes in the OS times of the patients. However, NO-BLP as a typical drug used in self-assembled LPHNPs enables easier entry into tumor cells by improving the tumor microenvironment.

In conclusion, although self-assembled LPHNPs, such as ABPLN, can increase the efficacies of chemotherapeutic medications by improving the tumor microenvironment, it is unclear whether self-assembled lipid nanostructures and self-assembled niosomes carrying different drugs can produce similar changes. Another open question is whether self-assembled lipid-based nanoparticles can change the tumor microenvironment by carrying different substances. In the future, such treatments must aim to target different types of tumors through various substances.

Lipid-based nanoparticles thicken the liposomes and increase their storage stability to a certain extent. Nevertheless, their ability to capture drugs remains to be improved. The utilization of organic solvents during preparation of specific lipid-based nanoparticles requires further research for reducing the toxicity of these nanoparticles. The present review clearly shows that data on animal and human experiments are lacking with regard to the use of nanomaterials in breast cancer chemotherapy.

This mini review summarizes recent research progress on lipid nanoparticles and provides detailed descriptions of the structures, mechanisms, and representative drugs associated with three types of self-assembled nanoparticles. Furthermore, we discuss the applications of lipid nanoparticles in the treatment of breast cancer.
